# Innovative functional juice enriched with native probiotics for enhanced nutrition and antimicrobial properties

**DOI:** 10.3389/fnut.2025.1552745

**Published:** 2025-02-19

**Authors:** Gabriela N. Tenea, Erika Perugachi

**Affiliations:** Biofood and Nutraceutics Research and Development Group, Faculty of Engineering in Agricultural and Environmental Sciences, Universidad Técnica del Norte, Ibarra, Ecuador

**Keywords:** maracuyá, coconut, native probiotics, antimicrobial activity, nutritional juice, antioxidant capacity

## Abstract

**Introduction:**

The increasing demand for consuming highly nutritional value foods based on fruits or vegetables in combination with “beneficial” lactic acid bacteria (LAB) expands the research on developing novel functional non-diary delivery vectors.

**Methods:**

In this study, the native *Lactiplantibacillus plantarum* UTNCys5-4 (Cys5-4) and commercial *Lactiplantibacillus plantarum* ATCC8014 (LP) strains have been targeted for probiotic properties in a novel designed tropical beverage containing yellow passionfruit (maracuyá) and coconut (MC). Furthermore, the quality of the newly fortified beverages, MCA (MC + Cys5-4) and MCB (MC + LP), was evaluated, along with their antimicrobial activity against two foodborne pathogens.

**Results:**

The results showed greater cell viability of Cys5-4 (8.84 ×10^7^ CFU/ml), whereas a statistically significant (*p* < 0.05) decrease was registered for LP strain (1.90 × 10^5^ CFU/mL) on the 15th day of storage with refrigeration. An enhanced ascorbic acid content (AAC) and total polyphenol content (TPC) in both fortified juices (MCA and MCB), with MCA showing moderate TPC increases and MCB showing slightly higher improvements. Both fortified juices exhibited increased antioxidant capacity (AOX) compared to the non-fortified control (MC), emphasizing their enhanced functional value. An intrinsic inhibitory activity against *Salmonella enterica* subsp. *enterica* ATCC51741 and *Escherichia coli* ATCC25922 was observed in the MC juice during the first 6 days of storage, after which the activity was lost. In contrast, the inhibitory activity in both MCA and MCB juices increased up to 15 days of storage, suggesting a synergistic interaction between the antimicrobial compounds produced by the bacteria and those naturally present in the fruit, effectively enhancing their role as *in situ* antimicrobial agents. These findings validate the use of Cys5-4 as a promising probiotic strain for developing functional beverages with improved shelf life, sensory properties, and health-promoting benefits.

**Conclusion:**

The synergistic antimicrobial and antioxidant effects underscore the potential of combining probiotics with tropical fruits like yellow passionfruit and coconut for innovative and nutritionally valuable non-dairy food products. These formulations present a promising solution for delivering native LAB strains, enabling the fruit and dairy industries to create healthier, market-driven products.

## 1 Introduction

The increasing demand for consuming highly nutritional value foods containing “special ingredients” such as probiotic bacteria expand the research on the identification of a suitable food bacterial carrier with improved properties. Traditionally, the delivery of probiotic bacteria is achieved via yogurt and other fermented dairy products (1). Nonetheless, several consumers suffer from lactose intolerance and probably do not have the benefits of these ingredients (2). Thus, searching for alternative raw materials as a delivery system for probiotic microorganisms remains of interest. Researchers are exploring fruit-based substrates as viable options, given their rich content of nutrients, vitamins, fiber, and antioxidants (3–6). However, the successful integration of probiotics into these substrates depends on the specific strain and the compatibility of the food matrix (7). Being positioned as a healthy food product and consumed frequently by a large percentage of the global consumer population, natural juices are among the essential constituents of the human diet (8). Some authors consider that producing bioactive peptides is an effective mode of antioxidative activity in foods containing probiotic bacteria (9). In addition, fruit juices have the advantage of showing a pleasant taste appreciated by all age groups and they are perceived as a healthy and different food. Early studies investigate the incorporation parameters such as inoculum, size, type (culture cells and lyophilized cells), pH, temperature as well as the organoleptic properties of different LAB strains in fruits matrices like, apple, bananas, oranges (10). Factors influencing the stability of probiotic microorganisms in the fruit matrices are the strain, the substrate composition, the acidity, the presence of antimicrobial substances, artificial dyes, and the production processes (11). However, the selection of a probiotic strain depends on its capacity to adapt (grow) in different food matrices and simultaneously produce antimicrobial substances that can protect the aliment from further contamination (12).

Tropical fruits are habitual commodities in many areas of South America, including Ecuador. Among them, yellow passionfruit (locally known as maracuyá) and coconut are widely advertised and usually consumed as juice. Despite their nutritional value and high production over the years, their use is limited due to their rapid deterioration. The poor storage conditions compromised fruit safety (13, 14). When sold under inadequate storage conditions, these fruits often pose safety concerns, becoming favorable environments for the growth of pathogenic microorganisms. Moreover, the application of beneficial bacteria like LAB, in the local market remains minimal, as most products rely on strains obtained from international collections (15). Consequently, there are currently no naturally derived beverages based on tropical fruits fortified with native probiotic bacteria available locally, nor documented in existing research. As the marketplace continues to demand more tropical fruits for natural juices, controlling the pathogen’s growth must be continuously monitored to prevent their safety (16). However, incorporating native probiotics as natural producers of antimicrobials in novel fruit-based matrices might be a useful alternative for food preservation along with the development of a novel food matrices to deliver beneficial ingredients. This approach will help expand the range of processed products in the local market, while also meeting the growing consumer demand for nutritious foods with health benefits. Previously, several LAB strains including *Lb. plantarum* Cys5-4 were selected as potential probiotic candidates *in vitro* (15, 17). In this study, we evaluated the effects of a novel functional beverage combining maracuyá (yellow passionfruit) and coconut juice (MC juice) on the cell viability of the native *Lb. plantarum* Cys5-4 (Cys5-4) strain, compared to the commercial *Lb. plantarum* ATCC 8014 (LP) strain to develop a new probiotic drink, with acceptable flavor and enhanced functional characteristics. We also examined the impact of juice matrices on cell viability during storage, alongside the sensory attributes (acidity, sweetness, flavor, and overall impression) to determine the optimal conditions for beverage production. Additionally, the study evaluated the antagonistic effects of each juice formulation, fortified with LAB strains, against two pathogenic strains, a property that could potentially extend the product shelf life. Finally, we determined the functional properties, including ascorbic acid content (AAC), total polyphenol content (TPC), and antioxidant activity (AOX), as well as technological properties such as pH, titratable acidity, and total soluble solids (TSS) throughout the storage period. By creating a novel maracuyá-coconut beverage enriched with native LAB strains, this innovation could offer a competitive edge in the market for functional products, providing characteristics that match or even surpass those of imported probiotic alternatives.

## 2 Materials and methods

### 2.1 Bacterial strain and growth conditions

The native strain *L. plantarum* UTNCys5-4 (GenBank accession number: KY041686.1) isolated previously from tropical *Malus* sp. fruits (Sucumbíos Provence of Ecuador), and the commercial strain *L. plantarum* ATCC8014 (LP) were used. The strains were grown in MRS broth medium (Difco, Detroit, MI, USA) at 35 ± 2°C under anaerobic conditions. The glycerol stocks 20% (v/v) were maintained at (−) 80°C and reactivated employing double passage on MRS-agar when needed.

### 2.2 Tropical juice formulation design and quality control

#### 2.2.1 Raw fruits and juice preparation

The maracuyá (*Passiflora edulis* Sims f. *flavicarpa* Deg.) and coconut (*Cocos nucifera*) fruits were purchased from the local market. The maracuyá fruits were chosen based on yellow kernel color, moderately wrinkled without any skin damage, while coconut was collected when the shell was a brown color and harder and the fruit meat was ready for consumption. After fruit disinfection with 0.16 hypochlorite solution, the kernel was removed, and the fruits were suspended in boiled water (90°C) for 5 min, then were grounded using an electric grinder (Oster, Milwaukee, WI, USA). For the preparation of MC juice, maracuyá, and coconut juice were independently prepared, and an equal proportion of each juice (v/v) was mixed with distilled water (twice as juice volume), the juice was pasteurized for 15 min at 85°C and filtrated with a four-fold gauze. After pasteurization, a heat shock on ice was applied to eliminate any harmful bacteria, and the juice was transferred to sterile glass bottles. The pH was determined using a pH device (Seven Compact S210, Mettler Toledo LCC, Columbus, OH, USA) and adjusted at 4.0 (with industrial sodium bicarbonate) before inoculation with bacteria. Bacteriological quality analysis of MC juice was performed using the plate count agar method to ensure that no harmful bacteria were presented before inoculation with probiotic bacteria (18). Independently, the juice aliquot was inoculated in MRS agar plates to detect the presence of fruit lactobacilli. Supplementary Figure S1A shows the process of juice formulation design.

#### 2.2.2 Fortification of juice with bacteria

For obtaining an initial cell density of 1 × 10^8^ CFU/mL in the final juice, 200 mL of 24 h culture of each bacterium was centrifuged at 5,600 × *g* for 5 min, the cells were washed twice with distillate water and the biomass was added into the juice (4.5 g cells/L juice). After inoculating each bacterium (Supplementary Figure S1B), a solution of 2% sterile glucose was added by mixing the bottles gently under the sterile flow chamber, the end of this procedure was considered day 0. The fortified juice matrices MCA (MC + Cys5-4) and MCB (MC + LP) were distributed in glass jars, 6 jars per each juice formulation (150 mL juice), tightly sealed and stored at 4°C (Supplementary Figure S1C). Non-inoculated juice (MC) was used as a control. The experiments were performed three times starting with a new batch of juice.

#### 2.2.3 Viable cells count determination during storage

Viable cells of both Cys5-4 and LP were determined at different intervals (0, 1, 3, 6, 9, 12, and 15 days) during storage at 4°C by the standard plate count method (Supplementary Figure S1D). Serial dilutions (with distillate water) of MCA and MCB juices were prepared. Aliquots of 0.1 mL of appropriate dilutions were plated in MRS agar (pH 6.2) plates and evaluated after incubation at 37°C for 48 h. Plates containing 20–350 colonies were recorded as colony-forming units (CFU) per mL of solution.

#### 2.2.4 pH, total titratable acidity (TTA), and total soluble solids (TSS) evaluation during storage

The pH levels were measured throughout storage using a pH meter (Seven Compact S210, Mettler Toledo LCC, Columbus, OH, USA). Total titratable acidity (TTA), expressed as a percentage of citric acid, was determined by titration with 0.01 N NaOH until reaching a pH of 8.2, following the method outlined in (19). Total soluble solids (TSS) were measured as Brix values in juice formulations with and without bacterial during 15 days of storage, using a refractometer (Reichert Arias OptiMatrix 500, Ametex BU, Germany). The MC juice without bacterial inoculation served as the control (Supplementary Figure S1D).

#### 2.2.5 Sensorial analysis

A trained panel of 20 participants, including laboratory staff, administrative personnel, and students from Universidad Técnica del Norte, assessed the sensory attributes of the juice formulations with and without bacterial addition (18). Each participant received 10 mL of each product in 100 mL glasses served at room temperature. They were asked to compare the formulations and provide feedback on sensory attributes, including acidic taste (with 5 points hedonic scale, where 5 represents the highest acidity and 1, no acidity), sweetness (with 5 points hedonic scale, where 5 represents the highest sweetness and 1, no sweet), maracuyá or coconut flavor (acceptable), and undesirable flavor such as vanished or rotten fruit odor (unacceptable) or off-flavors (no aroma). The overall sensory impression of the juices was recorded as either (−) not acceptable, (+) acceptable or indifferent.

### 2.3 Estimation of ascorbic acid content (AAC)

AAC was measured in the MCA, MCB, and MC juice formulations on days 1 and 15 of storage using the standard 2,6-dichloroindophenol titrimetric method (18). L-ascorbic acid (1 mg/mL) served as the standard, and the concentration was calculated relative to the standard, expressed in mg/L of juice. Additionally, Vitamin C levels were independently measured in both maracuyá juice and coconut juice, as well as their pasteurized and non-pasteurized mixtures (maracuyá: coconut, 1:1 v/v). The analyses were conducted in triplicate (Supplementary Figure S1D).

### 2.4 Determination of total phenolic content (TPC)

The TPC of the juice formulations (Supplementary Figure S1D) was assessed using the Folin–Ciocalteu method, with gallic acid (Sigma-Aldrich Co., Saint Louis, MO, USA) as the standard, following the procedure outlined in (19). To prepare the juice samples, 15 mL of each formulation (MCA, MCB, and MC) was treated with 35 mL of methanol and centrifuged at 8000 × g for 20 min. The supernatants were filtered through a 0.45 μm hydrophilic filter (ANPEL Scientific Instrument Co., Shanghai, China) and 500 μL was used for phenolic content analysis. Measurements were taken on days 1 and 15 of storage, with absorbance recorded at 760 nm using a spectrophotometer (Jenway 6,705 UV/Vis, Bibby Scientific Limited, UK). Results were reported as milligrams of gallic acid equivalents (GAE) per liter of juice. Triplicate analyses were conducted using freshly prepared juice batches. TPC was also assessed in pasteurized and non-pasteurized maracuyá and coconut juices, as well as their pasteurized and non-pasteurized mixtures (maracuyá: coconut, 1:1 v/v).

### 2.5 Determination of antioxidant activity (AOX)

The antioxidant activity (AOX) of the juice formulations (Supplementary Figure S1D) was evaluated on days 1 and 15 of storage using two radical scavenging assays: ABTS [2,2′-azino-bis(3-ethylbenzothiazoline-6-sulfonic acid)] and DPPH (1,1-diphenyl-2-picryl-hydrazyl). Both assays were performed following established protocols (20), using reagents sourced from Sigma-Aldrich Co., Saint Louis, MO, USA.

#### 2.5.1 ABTS radical scavenging activity

The juice extracts were prepared as described in Section 2.4. The ABTS^+^ cation radical was generated by reacting 7 mM ABTS solution with 2.45 mM potassium persulfate (Sigma-Aldrich Co. LLC, Saint Louis, MO, USA), and the mixture was stored in the dark at room temperature. The assay was conducted according to the method outlined by Re et al. (21), with minor modifications to calibrate the assay, the ABTS^+^ solution was diluted with methanol to reach an absorbance of 0.700 at 734 nm. For the test, 100 μL of juice extract from each formulation was mixed with 2.999 mL of the diluted ABTS^+^ solution. The absorbance was recorded after 30 min using a spectrophotometer (Jenway 6,705 UV/Vis, Bibby Scientific Limited, UK). The antioxidant activity of the juice samples was compared to a Trolox standard (Sigma-Aldrich Co. LLC, Saint Louis, MO, USA), and the results were expressed as Trolox equivalents (μmol Trolox/L of juice). The experiment was conducted in triplicate. Independently, ABTS activity was evaluated in pasteurized and non-pasteurized maracuyá juice, coconut juice, and their mixed juice formulations (1:1 v/v) under both pasteurized and non-pasteurized conditions.

#### 2.5.2 DPPH radical scavenging activity

The DPPH radical scavenging activity of the juice formulations, with and without bacterial fortification, was measured using a previously described (21). Briefly, 100 μL of juice extract from each formulation (collected on days 1 and 15 of storage) was mixed with 2 mL of 0.045 mg/mL methanolic DPPH solution. The mixture was incubated in the dark for 30 min, and the absorbance was measured at 517 nm using a spectrophotometer (Jenway 6,705 UV/Vis, Bibby Scientific Limited, UK). The antioxidant activity was compared to a Trolox standard, and the results were expressed as Trolox equivalents (μmol Trolox/L of juice). The experiment was performed in triplicate. DPPH activity was also evaluated in pasteurized and non-pasteurized maracuyá juice, coconut juice, and their mixed formulations (1:1 v/v) prepared under pasteurized and non-pasteurized conditions.

### 2.6 Antimicrobial activity of juice formulations during storage

The antimicrobial activity of the juice formulations was tested using the agar well diffusion method (15). *S. enterica* subsp. *enterica* ATCC51741 and *E. coli* ATCC25922 were used as indicator strains. Briefly, 100 μL of each indicator strain (7 log CFU/mL) was mixed with 1.5 mL of soft MRS agar (0.75%) and overlaid onto nutrient agar plates. After a 2-h incubation at 37°C, 500 μL of juice formulations, with or without added bacteria, was spotted into 12 mm diameter wells on the overlaid agar. The plates were incubated at 37°C, and zones of inhibition were measured after 48 h. The antimicrobial activity was assessed at various storage intervals (0, 1, 3, 6, 9, 12, and 15 days) using triplicate samples prepared from different juice batches (Supplementary Figure S1D).

### 2.7 Microbial safety of the final beverages

Microbiological safety, including the presence of total coliforms, Salmonella/Shigella, *E. coli*, yeasts, and molds, was assessed using the plate count agar method to ensure the absence of harmful microorganisms in the probiotic beverages (18). Total coliforms and *E. coli* were detected using Chromocult agar (Difco, Detroit, MI, USA) after 24 h of incubation at 37°C. Fungi and molds were identified using potato dextrose peptone agar following 5 days of incubation at 25°C. *Salmonella* spp. was enumerated using SS (Shigella-Salmonella) agar medium.

### 2.8 Statistical analysis

All experiments were conducted in triplicate, starting with independent juice batches. Results for cell viability were expressed as Mean CFU/mL ± Standard Deviation (SD). Data normality was evaluated using the Shapiro–Wilk test (48). One-way ANOVA followed by Tukey’s *post hoc* test was used to determine significant differences (*p* < 0.05) in pH, TTA, and cell viability results (SPSS 13.0, Inc., Chicago, IL, USA). Principal component analysis (PCA) was performed on four variables—cell viability, pH, TTA, and TSS—using the Spearman correlation matrix to assess changes during storage (days 1, 3, 6, 9, 12, and 15). Antimicrobial activity was analyzed using ANOVA with a split-plot design, followed by Duncan’s multiple range test and Least Significant Difference (LSD) with Bonferroni correction to evaluate significant differences between mean values.

## 3 Results and discussion

### 3.1 Evaluating the stability of probiotic LAB in a novel tropical juice formulation

The development of a next-generation bifunctional juice formulation with improved properties, including enhanced probiotic viability, antioxidant activity, antimicrobial efficacy, and pathogen inhibition, offers a promising strategy for delivering beneficial bacteria while simultaneously targeting harmful pathogens. The synergy between fruit matrix composition, metabolites, phenols, antioxidant compounds, and probiotic cells along with their active substances (i.e., antimicrobial peptides) represents a strategy to improve or restore gut health (22–24). The survival of probiotic microorganisms is obstructed by the acid conditions and food matrix composition, thus, is important to search for a suitable juice formulation that maintains or enhances the probiotic bacterial survival and improves the technological properties of the final product resulting in a pleasant and beneficial beverage. In this study, a natural fruit juice formulation based on maracuyá and coconut (MC juice) was tested as a new matrix for the delivery and survival of two LAB strains. The microbiological control analysis revealed that the MC juice does not contain any contamination (absence of coliforms, yeasts, molds) that could compromise its quality before fortification with the target LAB strain as no habitual LAB were detected in MRS-agar medium (data not shown). The changes in the cell viability population of Cys5-4 and LP during storage are shown in Table 1. Although a slight drop in cell counts was observed on day 1, the Cys5-4 adapted to the MC juice matrix survived during storage with refrigeration. The Cys5-4 cell counts registered on the 15th day of storage were greater than 8.84 × 10^7^ CFU/mL, thus above the threshold value (1.0 × 10^6^ CFU/mL) for consideration as a probiotic trait (22). Instead, a significant decrease (*p* < 0.05) in cell viability was observed in the MCB juice, the registered values on the 15th day being below (1.90 × 10^5^ CFU/mL) the probiotic threshold value suggesting that LP cells were sensitive to the new juice composition. The reduction of the cell viability might be linked with the metabolic activity including sugar consumption and acid production of the bacterial cell adapting to the new growth medium (e.g., juice), acid tolerance of bacteria is an important characteristic of a probiotic trait to survive during fermentation in a food matrix. Previous studies indicated a reduction of LP viability when inoculated in apple juice, indicating that the acidity of the juice affected its survival (25). Nagpal and co-workers (26) proved that orange and grape juices were suitable for probiotic *L. plantarum* and *L. acidophilus*, despite their high acidity. In other studies, the cantaloupe melon juice was found a suitable vehicle for the *L. casei* strain (22). Moreover, Lu and co-workers (23) demonstrated the successful probiotic fermentation of LAB strains in coconut water and durian pulp, respectively. In the present study, the Cys5-4 strain assent to the new juice formulation, suggesting that the survival might be related to its innate capacity to adapt and grow in this new matrix as the coconut juice provides more than 60% of dietary fiber exerting a prebiotic effect protecting or preserving the bacterial viability. On the other hand, maracuyá is a great source of vitamins (vitamins C and A), antioxidants, and fibers that contribute to the overall nutritional benefit of juice. Nonetheless, our results indicated that the probiotic viability is strain dependent and their survival is related to matrices composition and strain strength. The Cys5-4 adaptation to the new fruit beverage can be seen as a reflection of its ecological origin, apple fruit. The beverage matrix likely mirrors the bacteria’s natural habitat, facilitating a smooth transition and enabling rapid growth, fermentation, and metabolic activity (27). This adaptation highlights the ecological compatibility and resilience of the bacteria in fruit-based environments. The reduced adaptability of LP strain which is originating of corn silage to fruit matrices can be attributed to differences in nutrient availability, acidic pH, oxygen exposure, and the presence of plant-specific compounds (28). Additionally, the absence of specific enzymatic pathways, osmotic stress resistance, and competitive traits further limits their ability to thrive in fruit-based environments.

**Table 1:** Survival of bacterial cells in juices during storage with refrigeration.

Bacterial cell		UTNCys5-4	ATCC8014
Juice formulation		MCA	MCB
Cells viability (CFU/ml)	Time (days)		
	0	1.30 × 10^8^ ± 0.20 x 10^8a^	1.26 × 10^8^ ± 0.20 x 10^8a^
	1	9.50 × 10^7^ ± 0.50 × 10^7b^	5.40 × 10^7^ ± 0.20 x 10^7b^
	3	1.07 × 10^8^ ± 1.40 x 10^8ab^	5.20 × 10^6^ ± 0.5 x 10^6bc^
	6	1.10 × 10^8^ ± 0.50 x 10^8ab^	4.86 × 10^6^ ± 1.41 x 10^6c^
	9	1.35 × 10^8^ ± 0.50 x 10^8a^	3.60 × 10^6*^ ± 0.50 x 10^6d^
	12	9.30 × 10^7^ ± 1.35 x 10^7b^	1.60 × 10^6*^ ± 0.50 x 10^6e^
	15	8.84 ×10^7^ ± 1.35 x 10^7c^	1.90 × 10^5 *^ ± 0.50 x 10^5f^

The experimental values (means and standard deviations for *n* = 3). Different superscript letters indicate a statistically significant difference (*p* < 0.05) according to Duncan multiple test range. MCA, MC (maracuyá: coconut juice) with Cys5-4; MCB, MC (maracuyá: coconut juice) with LP; *, at this stage the juice was very acid, unpleasant and rotten aroma.

### 3.2 Physicochemical properties and sensory analysis of probiotic beverages during storage

**Figure 1:**
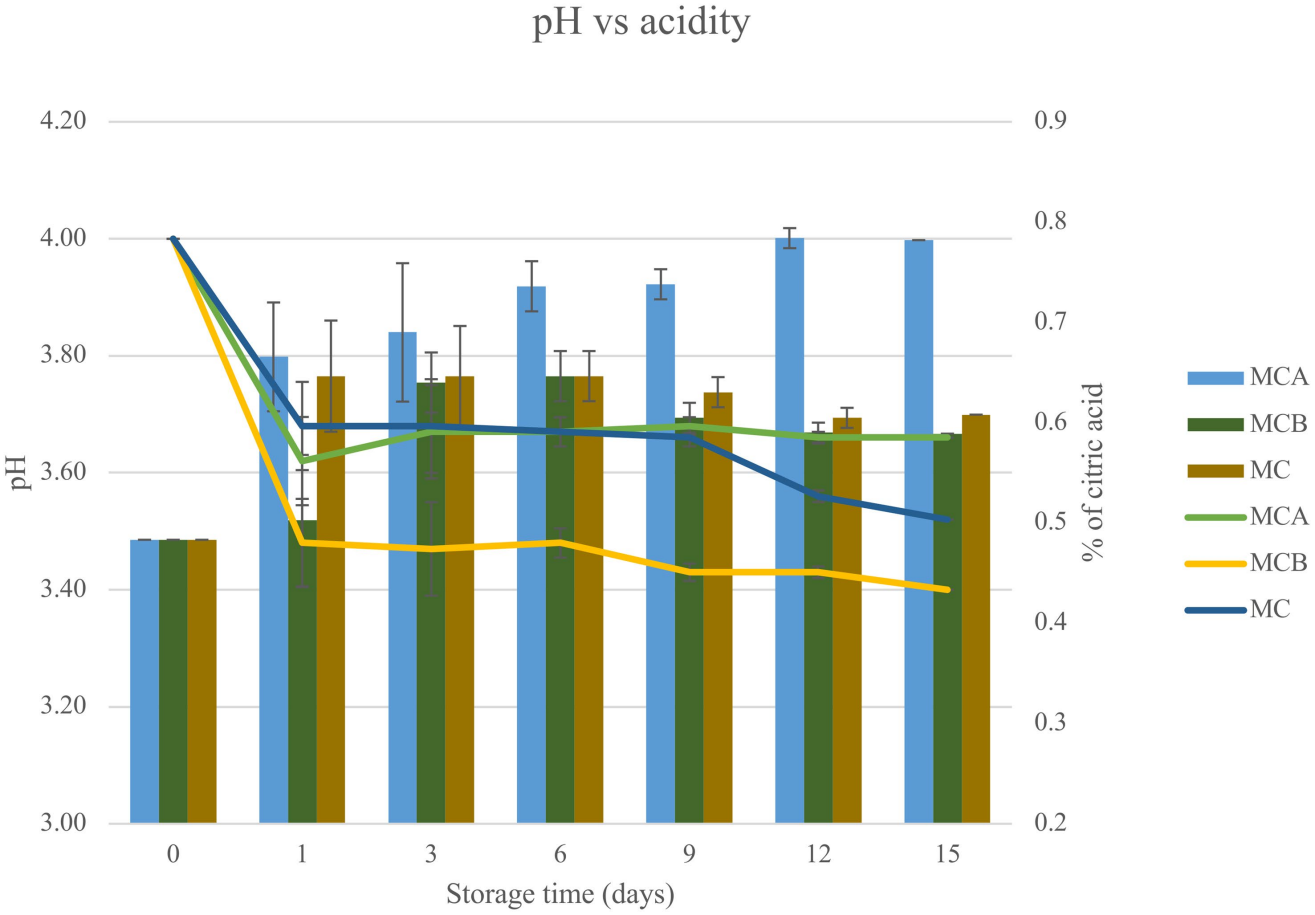
Bar chart represents the relationship between pH and acidity (measured as the percentage of citric acid) for three juice formulations (MCA, MCB, and MC) over a storage period of 15 days. The data presented in this figure are means of triplicate experiments ± SD. MCA, MC with Cys5-4; MCB, MC with LP; MC, maracuyá: coconut juice; color lines refer to pH and the bars represent the acidity.

**Figure 2:**
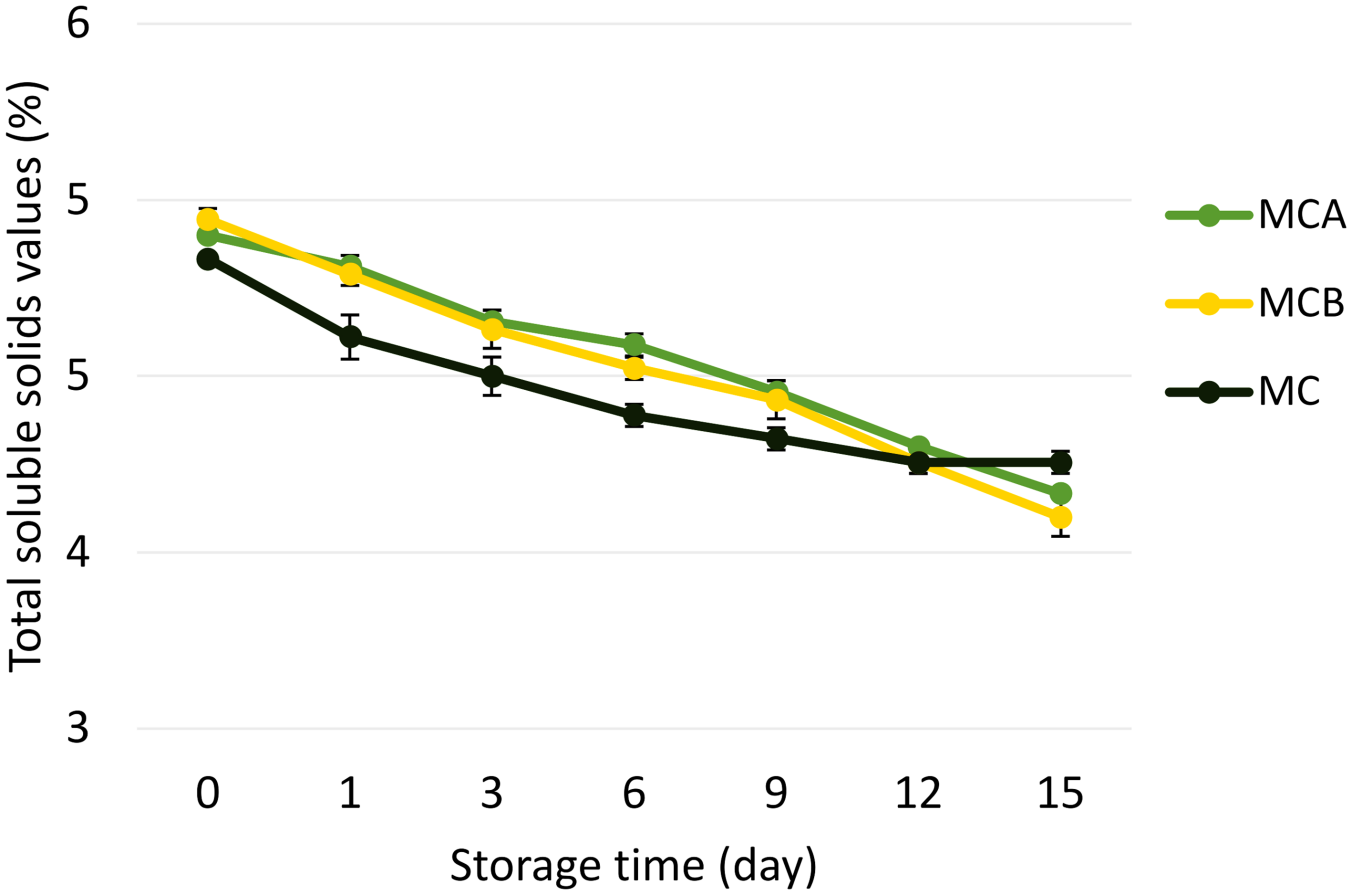
Changes in the total soluble solids content during storage. The data presented in this figure are means of triplicate experiments ± SD. MCA, MC with Cys5-4; MCB, MC with LP; MC, maracuyá: coconut juice.

**Figure 3:**
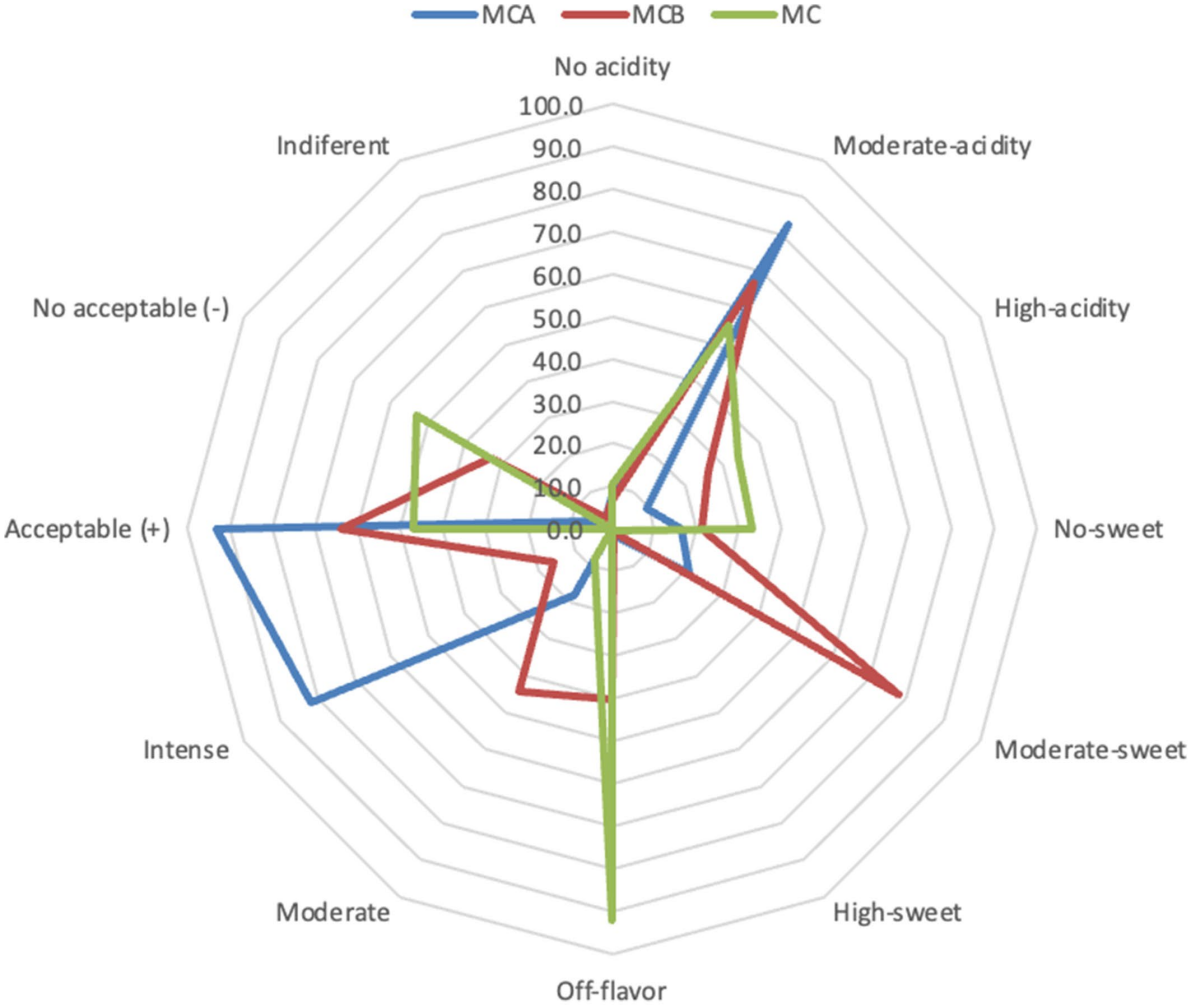
Radar plot of the sensorial analysis of the MCA, MCB and MC juices at the end of storage. MCA, MC + Cys5-4; MCB, MC+ LP; MC, maracuyá: coconut juice.

**Figure 4:**
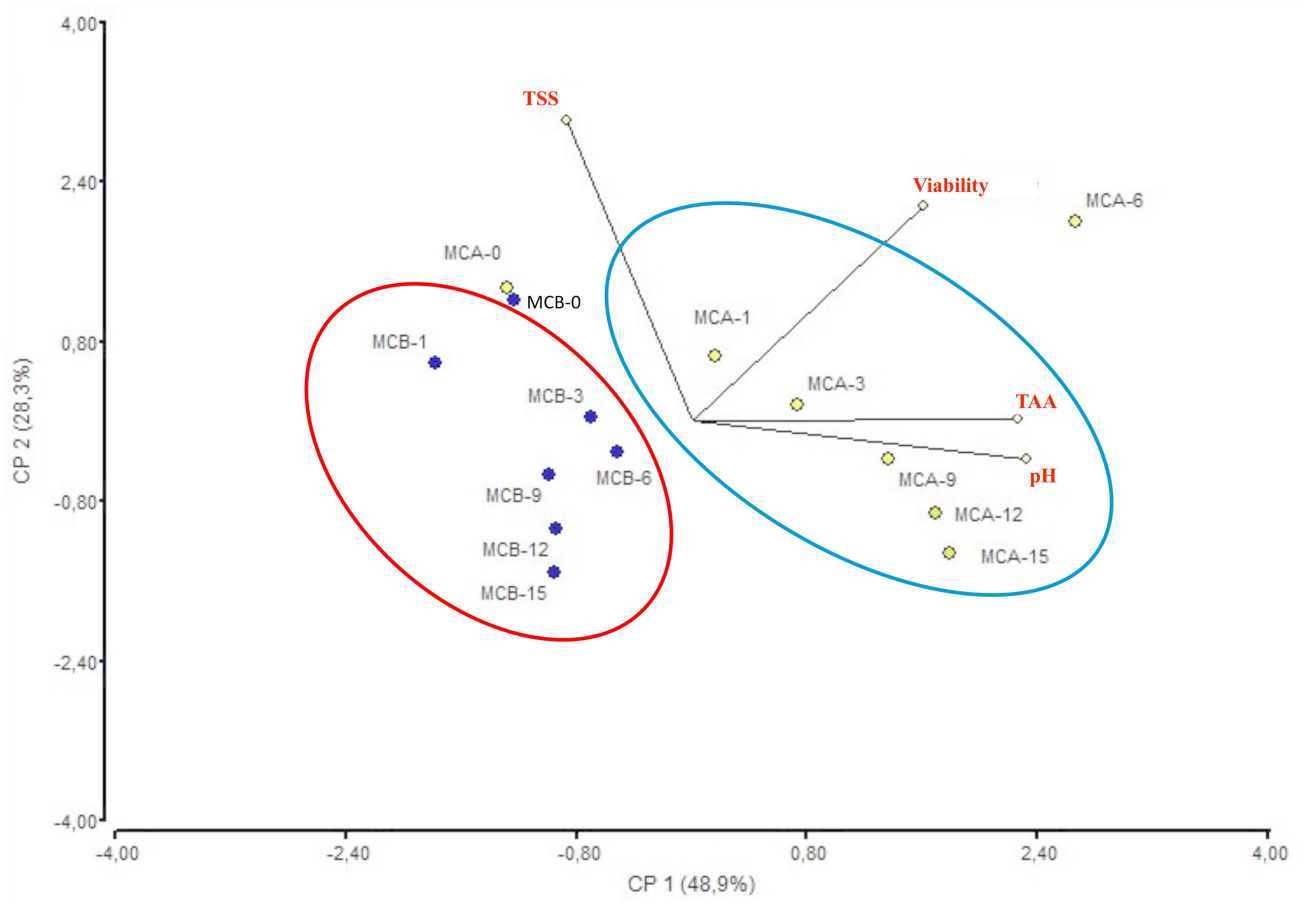
Biplot PCA analysis of the four variables (pH, TTA, TSS and cell viability) of MCA and MCB juices during storage. MCA, MC with Cys5-4; MCB, MC with LP; MC, maracuyá: coconut juice; TTA, total titratable acidity; TSS, total soluble solids.

A significant decrease in pH (*p* < 0.05) was registered after inoculation with bacteria (Figure 1). While at the inoculation point (day 0), the juice matrix had a pH of 4.0, on the 15th day of storage registered a significant reduction (*p* < 0.05) with a value of 3.66, 3.40, and 3.52 in MCA, MCB, and MC, respectively. The TTA was negatively correlated with the pH changes, the drop of pH during storage might be due to the increase of post-acidification capacity of probiotic bacteria, as the LAB remain active and continue to ferment available sugars in the juice, producing lactic acid and other organic acids as metabolic byproducts, which causes a gradual further drop in pH over time. The chemical composition of the juice, particularly the citric acid in maracuyá, may have also contributed to this process. Such changes were previously observed in a water coconut beverage inoculated with *L. rhamnosus* SP1 (29). The results suggest that the probiotic-fortified formulations (MCA and MCB) exhibit greater stability in terms of pH and acidity compared to the control juice (MC). The stability of MCA indicates that Cys5-4 strain may contribute to a more balanced fermentation process, maintaining sensory qualities and reducing excessive acidification during storage. This highlights its potential as a superior probiotic carrier in fruit-based juice formulations. Although a decline of TSS was recorded during storage, there were no significant differences (*p* > 0.05) between the juice formulation fortified with bacteria and the non-inoculated counterpart juice (Figure 2). The capacity of sugar consumption during storage relies upon LAB strain (29). In addition, to distinguish between the probiotic formulations and the conventional counterparts, it was necessary to establish the parameters for sensory characteristics such as acidity, aroma, or flavor being determinants of the overall impression of the new juice formulations (15). From day 1 upon bacterial inoculation, the MCA and MCB juices had different tastes and aroma than MC juice. The results demonstrated that the addition of bacterial strains to MC juice significantly influenced the sensory properties of the final product, with MCA juice being preferred for its taste and flavor over conventional MC or MCB juice (Figure 3). MCA juice had a dominant maracuyá aroma over coconut, which was appreciated by 93.3% of the panelists. Instead, the maracuyá aroma in MCB juice dissipate by day 9 of storage, leading to increased acidity resulting in dissatisfaction among panelists (data not shown). Additionally, the aroma of MC juice had faded entirely, replaced by a rotten fruit-like odor, high acidity, and an absence of the characteristic maracuyá fragrance, making it unappealing (Figure 3). In terms of sweetness, all juices were rated as moderate sweet aligning with the higher proportion of total solids detected in the samples (Figure 2). This result agreed with early investigation indicating that probiotics lead to considerable off-flavor in some products or had positive sensorial changes (perfume and sour) compared with the conventional juice samples without probiotic bacteria (30). In other studies, the sensory profile of apple beverage fermented with *L. casei* showed that the fermented product has a thick texture and sweet taste (31). Even so, MCA juice showed an enhanced fruit aroma during storage, while MCB and MC juice lost flavor (Figure 3). This might be related to the probiotic strain strength as the juice composition was similar. Based on this result, we concluded that Cys5-4, rather than LP, could be a promising option to enhance the flavor profile of juice over time, nonetheless, additional studies are required to identify the key compounds produced during storage with refrigeration. Moreover, a biplot was created using the PCA scores and factor loading to compare the similarities between the obtained juices with the two bacteria and understand the relationship between the physicochemical variables and the probiotic cell viability during storage. A PCA of four variables revealed a distinct separation between the MCA and MCB juice formulations (Figure 4). PC1 accounted for 48.9% of the total variance, while PC2 explained 28.3%. PC1 was predominantly associated with positive contributions from pH, titratable acidity (TTA), and cell viability, whereas PC2 was negatively correlated with total soluble solids (TSS). Spearman correlation values for the studied variables are presented in Table 2. A strong correlation (0.96) was observed between TTA and TSS, indicating that increased acidity (measured as % citric acid) corresponds to higher total solubility. Additionally, a direct correlation (0.70) between TTA and cell viability suggests that higher acidity supports probiotic viability. This highlights the positive effect of the juice matrix in maintaining the viability of probiotic strains, particularly Cys5-4 and LP, which are known to withstand lower pH levels—a critical factor for probiotic survival (18). A moderate correlation (0.56) was also identified between TSS and cell viability. Furthermore, pH and acidity exhibited a direct relationship, with a correlation value of 0.60, indicating that as acidity increases, pH decreases. Recent studies using *L. plantarum* UTNGt2 cells to fortify cacao-milk matrices demonstrated enhanced levels of vitamin C, total polyphenols, and antioxidant capacity (18). Based on these results, MCA is the superior formulation, maintaining higher pH, lower titratable acidity, and better bacterial viability during storage, which are critical for probiotic stability and functional benefits. This suggests that the conditions in these samples may favor probiotic survival and acidification. The distinct clustering of MCA and MCB samples suggests that they are influenced by different metabolic properties of inoculated bacteria in the juice matrix, leading to different physicochemical properties. This analysis underscores the efficacy of MCA as a carrier for probiotics, aligning with its enhanced physicochemical and microbiological stability over time compared to MCB.

**Table 2:** Spearman correlation coefficient to the four variables.

Variables	pH	TTA	TSS	Viability
pH	1	0.02	0.56	0.12
TTA	**0.60**	1	**0.96**	0.01
TSS	−0.17	−0.01	1	0.04
Viability	0.43	**0.70**	**0.56**	1

Values in bold are different from 0 with a significance level alpha = 0.05. TTA-total titratable acidity, TSS-total soluble solids, Viability: lactic acid bacteria cells viability.

### 3.3 Changes in the AAC of juices supplied with bacteria

LAB strains capable of fermenting tropical fruit juices into flavorful beverages with enhanced nutritional properties are of particular interest (32). L-ascorbic acid, commonly known as Vitamin C, is a key water-soluble compound with numerous biological and pharmaceutical functions (33). Its presence in fruit juices is beneficial not only nutritionally but also for lactic acid bacteria, as it acts as an oxygen scavenger while the nutrient-rich juice environment supports the growth of probiotic bacteria (34). The highest AAC (87.27 mg/L) was determined in maracuyá freshly unpasteurized juice, while the concentration was lower in coconut juice (3.71 mg/L) (Table 3). When mixed maracuyá with coconut, the AAC displayed a value of 48.56 mg/L while after pasteurization dropped significantly (5.1 mg/L). The effect might be attributed to the fact that vitamin C can be easily degraded by heat exposure (34). A significant increase in the AAC with 3.50 and 3.67-fold, respectively, was detected after the inoculation of probiotic bacteria in both MCA and MCB juices (Table 4). Although a little decrease was observed on the 15th day of storage with refrigeration in both fortified juices, the AAC was preserved in the probiotic juices, the decrease might be related to the traces of oxygen leading to its chemical oxidation (Table 4). Previous research indicated the influence of lactic acid fermentation on the content of vitamin C in fruit or vegetable juices (35). A decrease in AAC a few hours after the fermentation of juices based on sweet lemon, papaya, and prickly pear was detected (36). The fermentation of cashew apple juice by *Lb. plantarum* and *L. casei,* enhanced the AAC, while the use of *L. acidophilus* resulted in a decline of AAC by a few percent after 48 h of fermentation (37). The reduction of the ascorbic acid level after fermentation can be associated with an increased activity of the ascorbate oxidase enzyme produced by fermenting microbiota (37).

**Table 3:** Unpasteurized and pasteurized juice properties.

Sample	Treatment	AAC (mg Ascorbic acid/ L)	TPC (mg Gallic acid/ L)	AOX	
				ABTS (μMol Trolox/ L)	DPPH (μMol Trolox/ L)
Maracuyá juice	Unpasteurized (fresh)	87.27 ± 0.04	212.82 ± 0.16	5528.3 ± 0.01	4,240 ± 0.16
	Thermal pasteurized*	66.82 ± 0.04	292.82 ± 0.16	5370.0 ± 0.01	3,274 ± 0.16
Coconut juice	Unpasteurized (fresh)	3.71 ± 0.04	89.48 ± 0.16	1045.0 ± 0.01	3,531 ± 0.16
	Thermal pasteurized*	0.76 ± 0.04	37.23 ± 0.16	1303.4 ± 0.01	3,022 ± 0.16
MC juice	Unpasteurized (fresh)	48.56 ± 0.04	135.41 ± 0.16	2,920 ± 0.01	3,996 ± 0.16
	Thermal pasteurized*	5.1 ± 0.04	127.11 ± 0.16	1293.33 ± 0.01	3,990 ± 0.16

Values are mean ± standard deviation (*n* = 3). From three batches of juice. MC, maracuyá: coconut juice. AAC, acid ascorbic content; TPC, total polyphenol content; AOX, antioxidant activity; *, pasteurization conditions: 85°C, 3 min.

**Table 4:** Changes in AAC, TPC, and AOX of bacteria-fortified juice during storage with refrigeration.

Samples	Storage time (day)	AAC		TPC		AOX			
		mg Ascorbic acid/ L	% change vs control	mg Gallic acid/ L	% change vs control	ABTS method		DPPH method	
						μMol Trolox/ L	% change vs control	μMol Trolox/ L	% change vs control
MCA	1	17.82 ± 0.04	110.99	151.56 ± 0.01	17.55	3405.83 ± 0.01	132.55	5,235 ± 0.01	26.99
	15	14.22 ± 0.04	97.49	130.43 ± 0.01	10.13	2397.50 ± 0.01	62.53	4,901 ± 0.01	23
MCB	1	18.7 ± 0.01	114.29	190.08 ± 0.02	39.70	1851.67 ± 0.01	52.16	4,245 ± 0.01	6.19
	15	14.5 ± 0.04	98.97	144.52 ± 0.01	20.33	1772.50 ± 0.01	34.16	4,181 ± 0.01	7.21
MC	1	5.1 ± 0.05	(−)	127.11 ± 0.01	(−)	1293.33 ± 0.01	(−)	3,990 ± 0.01	(−)
	15	4.9 ± 0.05	(−)	117.85 ± 0.01	(−)	1255.83 ± 0.01	(−)	3,890 ± 0.01	(−)

MCA, MC + Cys5-4; MCA, MC+ LP; MC, maracuyá: coconut juice; AAC, acid ascorbic content; TPC, total polyphenol content; AOX, antioxidant activity, Values are mean ± standard deviation (*n* = 3) from three batches of juice. % change vs control was calculated as follows: % = [(V_1,15_T_1,15_-C_1,15_T_1,15_)/(average of V_1,15_T_1,15_ and C_1,15_T_1,15_)]*100, where V_1,15_T_1,15_ is the value of determined substance at time 1, 15 in the juice sample (MCA, MCB), C_1,15_T_1,15_ is the value of determined substance in the control juice (MC) at time 1, 15 of storage.

### 3.4 TPC and AOX variation during storage

One of the major contributors to the antioxidant activity of fruit or vegetable juices is their content of phenolic compounds (38). Previous research indicated that in potato juice, the total phenolic content was dependent on fruit variety, and a reduction of TPC was reported in cabbage juice (39). The TPC in maracuyá fresh unpasteurized juice was 212.82 mg gallic acid/L, while in the coconut juice fresh and unpasteurized was 89.48 mg/L (Table 3). Contrary, in the unpasteurized MC juice, the TPC was 135.41 mg gallic acid/L, while a 35.45% decrease was detected upon the heat treatment. The fortification of juices with bacteria resulted in the restoration of TPC with a significant increase of 10.13% in MCA and 20.30% in MCB at the end of storage (Table 4). The AOX of maracuyá independently fresh-made pasteurized juice was 2.86% lower than its unpasteurized counterpart by both ABTS and DPPH methods, whereas in pasteurized coconut the antioxidant activity enhanced with 24.72% according to the ABTS method (Table 3). We hypothesized that the enhanced antioxidant activity after heat exposure might follow the same pathway as a heat-treatment-induced chemical reaction between active components as amino- and carbonyl- groups, known as the Maillard Reaction (40). Our data agreed with previous studies indicating that the pasteurization condition might increase the polyphenolic content and antioxidant activity (41). Although the TPC dropped from initial to final storage in both MCA and MCB juice formulations, the yield was superior with 17.55 and 39.70% respectively, rather than its MC counterpart. Additionally, pasteurization (MC) had no significant impact on the total antioxidant capacity of the juice matrix during storage, as assessed by both ABTS and DPPH assays (Table 3). This indicate that the storage did not compromise the juice’s antioxidant properties, and fortification with probiotic bacteria significantly (*p* < 0.05) enhanced antioxidant capacity. The MCA juice exhibited the most notable improvement, with a 62.53% increase, while MCB juice demonstrated a 34.16% rise in antioxidant activity by the end of the storage period (Table 4). Consistent findings were observed using the DPPH method, where fortification with Cys5-4 and LP strains led to significant (*p* < 0.05) improvements in AOX compared to unfortified MC juice. Despite this, the LP strain’s decreased cell viability below the probiotic threshold at the end of storage highlights the influence of the juice matrix on probiotic efficacy. Previous studies have shown that *L. plantarum* strains enhanced the antioxidant capacity of fruit and vegetable juices, such as mulberry juice and pineapple juice, when fermented with *Weissella cibaria* 64 and *Leuconostoc mesenteroides* 12b (42–44). Similarly, the AAC, TPC, and AOX values in the fortified maracuyá-coconut juice gradually declined during storage but remained superior to the unfortified MC juice. This study represents the first report demonstrating improved antioxidant activity and ascorbic acid content in maracuyá-coconut juice fortified with a native probiotic strain, offering a novel, high-quality functional beverage with enhanced health benefits.

### 3.5 Cys5-4 cells exhibit strong pathogen-inhibitory effects against *Salmonella enterica* and *Escherichia coli* in the prepared juices

**Figure 5:**
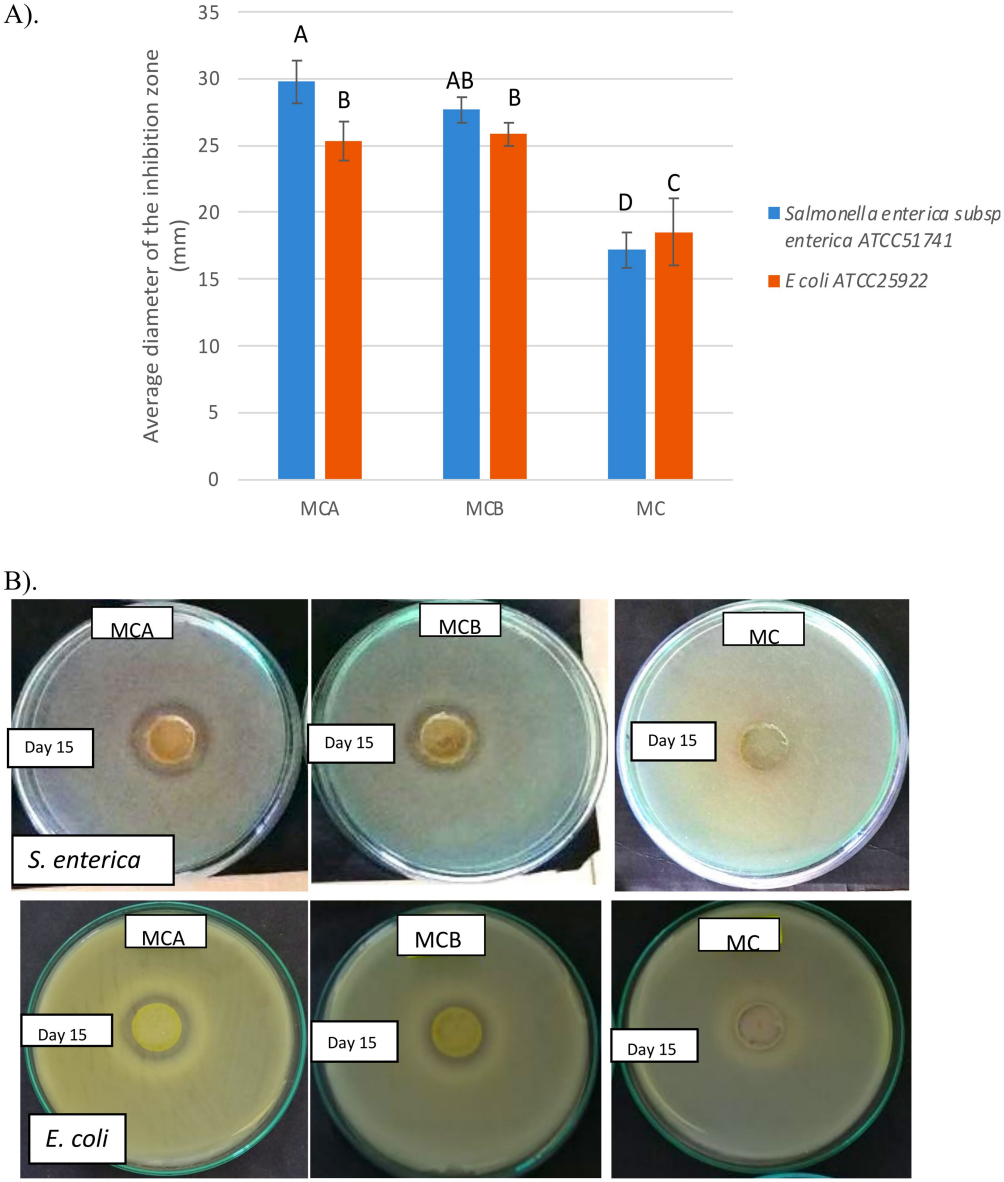
**(A)** Antimicrobial activity of juices formulation toward *Salmonella enterica* subsp. *enterica* ATCC51741 and *E. coli* ATCC25922. **(B)** The image shows the inhibition halo formed against indicator strains on the 15th day of storage. MCA, MC + Cys5-4; MCB, MC+ LP; MC: maracuyá: coconut juice Bars represent the means and standard deviations for *n* = 3, Values with different letters are significantly different *p* < 0.05.

Antimicrobial compounds produced by probiotic bacteria are a valuable feature for suppressing the growth of harmful microorganisms in food, thereby preventing spoilage and extending shelf life (45). The antimicrobial activity of the Cys5-4 cell-free supernatant in orange juice has been previously documented (15), further supporting its potential as a natural preservative. In this study, the inhibitory activity of all juice formulations against *S. enterica* ATCC51741 and *E. coli* ATCC25922 was evaluated during storage. Figure 5 shows the comparative antimicrobial activity of MCA, MCB, and MC matrices, expressed as average inhibition zones (mm). A significant difference (*p* < 0.05) in inhibitory activity was observed between the fortified juices (MCA and MCB) and the unfortified MC juice. While MC juice exhibited innate antimicrobial activity from day 1 to day 6, likely due to its low pH (3.31) or active fruit metabolites, this activity diminished after day 6. In contrast, MCA and MCB juices maintained and even slightly enhanced inhibitory activity throughout storage, particularly in MCA, suggesting that antimicrobial effects stem from bacterial components released into the juice or a synergistic interaction between bacterial metabolites and the bioactive compounds of the fruit. The stronger inhibition observed in MCA juice compared to MCB, particularly against *Salmonella*, highlights the strain-specific nature of the antimicrobial effects. Previous studies have demonstrated similar findings, such as the antimicrobial activity of maracuyá leaf and stem extracts against Gram-positive and Gram-negative bacteria (46), maracuyá seed extract against *Propionibacterium acnes* (45), and fermented sweet lemon juice exhibiting stronger antimicrobial activity than non-fermented versions (34). These findings corroborate earlier research showing that Cys5-4 can survive in diverse matrices, such as raw meat, while exerting antagonistic effects against co-existing bacteria (47). The biological activity of Cys5-4 remained intact during storage, underscoring its potential as a multifunctional strain in newly designed juice formulations. Developing probiotic tropical juice products that inhibit pathogenic bacteria while maintaining quality and functional properties offers significant promise for the food industry. This study underscores the promise of maracuyá-coconut juice as a vehicle for delivering probiotic strains with antimicrobial properties and health-enhancing benefits, opening new avenues for the development of innovative functional drinks.

## 4 Conclusion

This study shows that fortifying maracuyá (yellow passionfruit) and coconut juice with a native probiotic results in the development of a novel beverage with favorable physicochemical properties and sensory characteristics. The novel formulation maintained the probiotic viability during storage and showed inhibitory capacity toward foodborne pathogens. Supplementing maracuyá-coconut juice formulations with Cys5-4 has a minimal impact on physicochemical quality while significantly enhancing bioactive compounds (TPC, AOX, and AAC) (*p* < 0.05) compared to the control juice without bacterial addition. These findings underscore the synergistic benefits of combining the juice’s rich nutritional profile—vitamins, minerals, and bioactive compounds—with the functional properties of probiotics, offering a promising strategy for developing health-enhancing beverages. However, the challenge of identifying optimal food matrices to support biologically active strains remains a key focus for advancing next-generation probiotic products. Future research should focus on examining the metabolic changes in fortified juices and investigating enzymatic processes, such as polyphenol hydrolysis, to enhance and refine the functional properties of these innovative probiotic-enriched beverages. These studies will support the way for crafting functional beverages that meet consumer desires for nutritious, high-quality products enriched with additional health benefits.

## Data Availability

The raw data supporting the conclusions of this article will be made available by the authors, without undue reservation.
