# Supplementary material for: Innovative functional juice enriched with native probiotics for enhanced nutrition and antimicrobial properties
Source: Front Nutr. 2025 Feb 19;12:1552745. doi: 10.3389/fnut.2025.1552745 (PMC11879804; doi:10.3389/fnut.2025.1552745)
Supplement: Supplementary file 1 [file Data_Sheet_1.docx]

**Supplementary files**

**Innovative Functional Juice Enriched with Native Probiotics** **for Enhanced Nutrition and Antimicrobial Properties**

Gabriela N. Tenea *, Erika Perugachi

Biofood and Nutraceutics Research and Development Group, Faculty of Engineering in Agricultural and Environmental Sciences, Universidad Técnica del Norte. 100150 Ibarra, Ecuador.

*** Correspondence:**Corresponding Author: Gabriela N. Tenea
gntenea@utn.edu.ec

**Figure S1.** Workflow of the juice manufacturing. A. Fruits processing; B. bacterial biomass preparation; C. Bottling and storage; D. product analysis

**
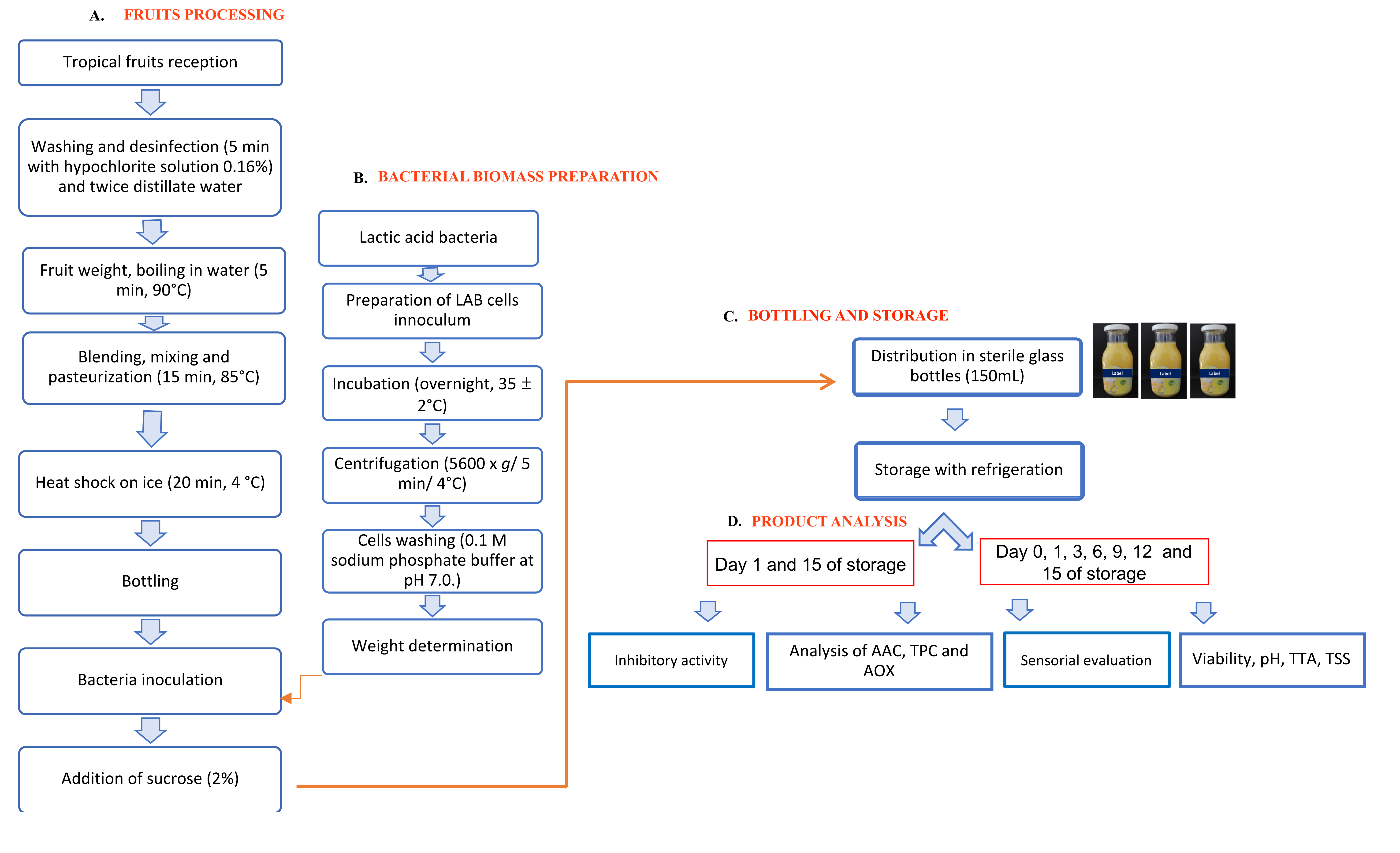
**
